# Effects of Caffeic Acid Phenethyl Ester on Embryonic Development Through Regulation of Mitochondria and Endoplasmic Reticulum

**DOI:** 10.3390/vetsci11120625

**Published:** 2024-12-06

**Authors:** Chu-Man Huang, Hui-Mei Huang, Ying-Hua Li, Xing-Wei Liang, Nam-Hyung Kim, Yong-Nan Xu

**Affiliations:** 1 Guangdong Provincial Key Laboratory of Large Animal Models for Biomedicine, South China Institute of Large Animal Models for Biomedicine, School of Pharmacy and Food Engineering, Wuyi University, Jiangmen 529000, China; 18318843832@163.com (C.-M.H.); h2914095135@163.com (H.-M.H.); yhli@wyu.edu.cn (Y.-H.L.); 2 College of Animal Science & Technology, Guangxi University, Nanning 530004, China; xwliang@gxu.edu.cn

**Keywords:** caffeic acid phenethyl ester, embryonic development, mitochondrial function, endoplasmic reticulum

## Abstract

Caffeic acid phenethyl ester (CAPE) is one of the main active components of the natural medicine propolis, which has antioxidant, anti-tumor, and immunomodulatory activities. This study aimed to analyze the effects and underlying mechanisms of CAPE added to the medium of in vitro cultures on the developmental competence, mitochondria, and endoplasmic reticulum of porcine embryos. The results demonstrated that 1 nM of CAPE significantly improved the quality of porcine embryos, increased the rate of blastocyst formation, and enhanced the proliferation ability. It also enhanced mitochondrial function by increasing the level of mitochondrial membrane potential and expression of the mitochondrial biogenesis-related protein PPARgamma coactivator 1 alpha and beta (PGC1 alpha and beta), regulating mitochondrial biogenesis, and increasing adenosine triphosphate (ATP) content. In addition, CAPE alleviated oxidative and endoplasmic reticulum (ER) stress in embryos by decreasing ROS accumulation and increasing glutathione content, as well as elevating Nrf2 and reducing GRP78 (ER stress marker) expression levels. Moreover, CAPE reduced the levels of apoptosis and autophagy in the cultivated embryos. These results indicate that CAPE improves the quality and enhances the mitochondrial function of in vitro-produced porcine embryos by alleviating oxidative and ER stress.

## Simple Summary

In this experiment, porcine embryos were used as research subjects, and the effects of caffeic acid phenethyl ester on the embryos’ quality and the mitochondrial function were evaluated by examining the quality and developmental ability of blastocysts, the antioxidant properties of embryos, and the mitochondrial function, endoplasmic reticulum stress level, autophagy, and apoptosis.

## 1. Introduction

The increasing application of advanced reproductive technologies, including transgenics, animal cloning, in vitro fertilization, and embryo production, necessitates high-quality embryos [1].

Reactive Oxygen Species (ROS) represent a variety of derivatives of molecular oxygen formed as inevitable byproducts of cellular processes. They function as signaling molecules at physiological levels and originate from the activity of metabolic pathways, cytokine/growth factor receptors, and, primarily, mitochondria. Oxidative phosphorylation in mitochondria fuels processes such as gametogenesis, fertilization, and early embryonic development, as well as ROS formation by an electron transfer to the molecular oxygen [2,3]. In addition to producing ROS, mitochondria also generate cellular energy, regulating cell growth, information transfer, and apoptosis [4,5,6,7]. Therefore, maintaining mitochondrial homeostasis is crucial for successful mammalian reproduction. In vitro embryo culture exposes embryos to higher oxygen concentrations than their in vivo environment, causing ROS accumulation. As a result, abnormal ROS levels may provoke antioxidant barrier disruption, oxidative stress, apoptosis, mitochondrial dysfunction, and autophagy [8,9], compromising embryo quality in vitro.

Propolis is a natural resinous mixture collected by bees and utilized for centuries in traditional medicine due to its diverse therapeutic properties [10,11]. Its chemical complexity includes caffeic acid phenethyl ester (CAPE), which has antioxidant, antiviral, anti-inflammatory, anticancer, and immunomodulatory properties, as well as hypoglycemic, cardioprotective, and gastric secretory inhibition functions. Moreover, CAPE acts as a specific inhibitor of NF-κB (nuclear factor kappa-B) [12,13], which is an essential regulator of the immune response to infection. This compound also significantly improves sperm quality parameters and enhances the antioxidant capacity of spermatozoa by increasing the activity of catabolic enzymes, such as catalase (CAT), superoxide dismutase (SOD), and glutathione peroxidase (GPX), as well as by modulating pathways involving AMP-activated protein kinase (AMPK), caspase 3 (CASP3), and SOD [14]. In an in vitro model of local ischemia, CAPE mitigates renal mitochondrial damage, improves oxidative phosphorylation, enhances mitochondrial Ca^2+^ uptake, blocks ischemia-induced CASP3 activation, and protects renal cells from necrosis [15]. Furthermore, given the intricate functional interactions between the mitochondria and endoplasmic reticulum (ER), CAPE attenuates doxorubicin-induced ER dysfunction in H9c2 embryonic rat cardiac cells [16].

Because CAPE significantly increases the antioxidant capacity of spermatozoa and has beneficial properties for the mitochondrial and ER functions in H9c2 cells, we hypothesized that it would confer similar effects to porcine embryos. Accordingly, this study aimed to reveal the effects and underlying mechanisms of CAPE on the porcine embryo quality by assessing their mitochondrial function, ER and oxidative stress, and cell death. Our results provide a reference to optimize the medium for in vitro embryonic development and further rationalize using CAPE as a drug.

## 2. Materials and Methods

All chemicals and reagents were purchased from Sigma-Aldrich (St. Louis, MO, USA), unless otherwise stated.

### 2.1. Oocyte Collection and In Vitro Maturation (IVM)

Ovaries from adolescent sows, collected at the slaughterhouse in Jiangmen City, China, were stored in thermos flasks and utilized in the laboratory for the duration of the experiments. The ovaries were washed 4× at 37.5 °C to keep the surface clean. Using the syringe, the cumulus–oocyte complexes (COCs) can be obtained and placed into a pre-warmed working solution (TL-HEPES). At 37.5 °C, the COCs were allowed to precipitate under gravity over time. The supernatant was aspirated and discarded, resulting in clean COCs without impurities. A TCM-199 maturation medium (Thermo Fisher Scientific, #11150-059, Waltham, MA, USA) supplemented with 10% porcine follicular fluid, 0.57 mM L-cysteine, 20 ng/mL epidermal growth factor, 1% penicillin–streptomycin (Thermo Fisher Scientific), 0.2 mM sodium pyruvate, and 10 IU/mL follicle-stimulating hormone and luteinizing hormone (Ningbo No. 2 Hormone Factory, Ningbo, China) was used as the IVM medium. The well-encapsulated COCs in the cumulus were picked out, washed with TL-HEPES and IVM medium, 100 per pool, and immediately immersed into 0.5 mL of IVM medium and incubated for 44–46 h at 5% CO_2_, 37.5 °C.

### 2.2. Parthenogenetic Activation and In Vitro Embryo Production

The activation solution and IVC medium (bicarbonate-buffered PZM-5 supplemented with 4 mg/mL bovine serum albumin) need to be placed in an incubator before use, wherein the activation solution is made up of 300 mM mannitol, containing 0.5 mM HEPES, 0.05 mM CaCl_2_, 0.1 mM MgSO_4_, and 0.01% polyvinyl alcohol. The COCs were blown through a working solution containing 1% hyaluronidase to strip off the outer cumulus layers. Parthenogenetic activation of naked oocytes was performed with two 120 V, 60 μs DC pulse currents in the activation solution. Activated oocytes were incubated 3–4 h in IVC medium containing 7.5 mg/mL cytochalasin B to inhibit the excretion of dipolar bodies. Finally, embryos were transferred to IVC medium for 7 days at 37.5 °C, and with 5% CO_2_, in a saturated humidity incubator.

CAPE was dissolved to a higher concentration with dimethyl sulfoxide (DMSO) and diluted to the desired working concentration with IVC, wherein the amount of DMSO was approximately 0.000001% (<0.025%). Embryos were cultured in normal IVC medium without additional additives for seven days at 37.5 °C and 5% CO_2_ as a control group (Con). Based on previous pilot experiments, we found that the nM concentration level of CAPE had a better effect on the blastocyst rate, and performed more consistently than the uM concentration, especially since a slightly higher concentration is prone to reverse the inhibition of embryo growth. Accordingly, embryos were cultured in the IVC medium containing different concentrations of CAPE (0, 0.1, 1, or 20 nM) to determine the CAPE dose suitable for early porcine embryos. The optimal concentration was inferred according to the rate of blastocyst formation.

### 2.3. Determination of Changes for Glutathione (GSH) and ROS Levels

Detection of intracellular ROS or GSH levels was achieved with 10 μM 2′,7′-dichlorodihydrofluorescein diacetate (DCFH-DA; Beyotime Biotechnology, Shanghai, China) or 10 μM 4-chloromethyl-6,8-difluoro-7-hydroxycoumarin (CMF2HC; Invitrogen, Rochester, NY, USA). In brief, washed 4-cell-stage embryos were placed into dye at 37.5 °C for half an hour each, washed 4×, and imaged using a fluorescence microscope (Ti2eU; Nikon, Tokyo, Japan). The corresponding fluorescence intensity was assessed using ImageJ version 8.0.2 software (NIH, Bethesda, MD, USA).

### 2.4. Mitochondrial Distribution and Membrane Potential Assays

In brief, 4-cell-stage embryos were placed into 10 μM 5,5′,6,6′-tetrachloro-1,1′,3,3′-tetraethylbenzimidazolylcarbocyanine iodide (JC-1; Invitrogen) dye and 200 nM MitoTracker Red CMXRos (#C1049B; Beyotime Biotechnology, Shanghai, China) for 30 min each. Washed embryos were imaged under a fluorescence microscope (Ti2eU; Nikon, Tokyo, Japan), and the corresponding fluorescence intensity was analyzed with ImageJ version 8.0.2 software (NIH, Bethesda, MD, USA). The average mitochondrial membrane potential (MMP) was calculated as the ratio of red to green fluorescence intensity.

### 2.5. Determination of ATP Content

For mitochondrial function evaluation, ATP levels were measured in embryos with an ATP assay kit (#S0026; Beyotime Biotechnology, Shanghai, China). In brief, washed day 7 blastocysts were pooled and lysed in cell lysis buffer as soon as possible. The 20 best blastocysts were selected for each group to ensure consistency between samples. The ATP standard solution was diluted to the appropriate concentration gradient by an ATP standard diluent. A 96-well plate was filled with 100 µL of ATP-detection working solution per well, and the standard curve was plotted using multifunctional microplate fluorescence emission values. The ATP detection lysates and reagent were added to each of the detection holes, and the luminescence was measured using an automatic microplate reader (Biotek Synergy Neo2, Agilent Technologies, Santa Clara, CA, USA). It is worth noting that all steps should be conducted on ice.

### 2.6. Proliferation Assay

The BeyoClick EdU-647 Cell Proliferation Detection Kit (Beyotime Biotechnology, Shanghai, China) was used for this experiment. In brief, blastocysts were incubated in the medium containing 10 μM EdU for 8 h, followed by washing with PBS-PVA. Blastocysts were fixed in 3.7% paraformaldehyde, and then permeabilized in 0.1% Triton X-100, both at room temperature for thirty minutes. After that, the blastocysts were washed 4× and fixed in 1% bovine serum albumin (BSA) for 1 h. They were promptly washed in PBS-PVA and incubated in darkness with the BeyoClick additive solution at room temperature for 1 h. Blastocysts were then incubated in darkness with 10 µg/mL Hoechst 33342 at 37.5 °C for 15 min to stain the nuclei. The number of EdU-positive cells and the total cell number were determined using a fluorescence microscope (Ti2eU; Nikon, Tokyo, Japan). The proliferation rate was calculated as the ratio of EdU-positive cells to total cell number.

### 2.7. TUNEL Assay

After being fixed and permeabilized, washed blastocysts were fixed in 1% BSA for 1 h. They were promptly washed again and incubated in darkness with fluorescein-conjugated dUTP and a terminal deoxynucleotidyl transferase enzyme (In Situ Cell Death Detection Kit, Roche Diagnostics, IN, USA) at 37.5 °C for 1 h. Afterwards, the blastocysts were incubated in darkness with 10 µg/mL Hoechst 33342 for 15 min to stain the nuclei. The number of apoptotic nuclei and the total cell number were calculated with a fluorescence microscope (Ti2eU; Nikon, Tokyo, Japan). Apoptosis was evaluated according to the percentage of apoptotic nuclei.

### 2.8. Immunofluorescence Staining

After being fixed and permeabilized, blastocysts were washed 4× and fixed in 1% BSA for 1 h. Afterwards, blastocysts were incubated overnight at 4 °C with the following primary antibodies: anti-LC3B (1:200; #ab48394, Abcam, Cambridge, MA, USA), anti-Nrf2 (1:200; #ab31163, Abcam), anti-PGC1 alpha and beta (1:200; #ab72230, Abcam), and anti-GRP78 BIP (1:200; #ab21685, Abcam). Further, the washed blastocysts were incubated for 1 h with goat anti-rabbit IgG (1:500; #ab150077, Abcam) or anti-rabbit IgG (H+L) (1:500; #8889, Cell Signaling Technology, Danvers, MA, USA) secondary antibodies. The final incubation was completed with 10 µg/mL Hoechst 33342 in darkness at 37.5 °C for 15 min to stain the nuclei. The corresponding fluorescence intensity was assessed and calculated with a fluorescence microscope (Ti2eU; Nikon, Tokyo, Japan) and ImageJ version 8.0.2 software (NIH, Bethesda, MD, USA).

### 2.9. Quantitative Reverse Transcription-Polymerase Chain Reaction (RT-qPCR)

Blastocysts were collected from the medium, and the total RNA was extracted from a pool of 20 blastocysts using the Dynabeads mRNA DIRECT Purification Kit (Invitrogen). The purified RNA was reverse transcribed to cDNA using a reverse transcription kit (Tiangen Biotech, Beijing, China). The KAPA SYBR FAST qPCR Master Mix (2×) Kit (KAPA Biosystems, Roche, Basel, Switzerland) was used to prepare 20 µL qPCR reactions, with each containing 1 µLcDNA template, as well as forward and reverse primers. The qPCR assays were performed in triplicate in a LightCycler 96 (Roche), as follows: initial denaturation, 3 min at 95 °C; 40 cycles of 3 s at 95 °C (denaturation), 30 s at 60 °C (annealing), and 20 s at 72 °C (extension); and the final extension, 30 s at 37 °C. The melting profiles, analyzed in pre-experiments, had a single peak and were within a reasonable temperature range. Furthermore, 18S rRNA is present in the small subunit of ribosomes in all eukaryotic cells, and its encoding gene RN18S is quite conserved during biological evolution, thus being more stable and less affected by RNA degradation than other internal reference genes. According to the existing studies, it has been demonstrated that 18S rRNA expression is large and stable during zebrafish embryonic development [17]. Hence, gene expression levels were quantified using the 2^−ΔΔCt^ method, with RN18S as the internal control. The annealing temperature of all reactions was 60 °C. All primers used in the assays are shown in Table 1.

### 2.10. Statistical Analysis

Each biological experiment was repeated at least 3 times. Data were presented as the mean ± standard error mean (SEM). The t-test is a statistical method used to compare the differences between two independent samples that are independent of each other and do not interfere with each other. One-way ANOVA (Tukey–Kramer) is used to test the significance of the difference between the means of two or more samples. Both methods are used to test whether the sample means are significantly different, and both require the data to be normally distributed. Hence, statistical differences between 2 groups were calculated with the t-test, and those between 4 groups were determined with one-way ANOVA. Statistical analyses were performed using SPSS (version 22.0, IBM Corp, IBM Corp, Chicago, IL, USA), including checking for the normality of the variables. Results were significant when * *p* < 0.05, ** *p* < 0.01, or *** *p* < 0.001.

## 3. Results

### 3.1. Effects of Different CAPE Concentrations on the Formation of Early Embryonic Blasto-Cysts In Vitro

**Figure 1:**
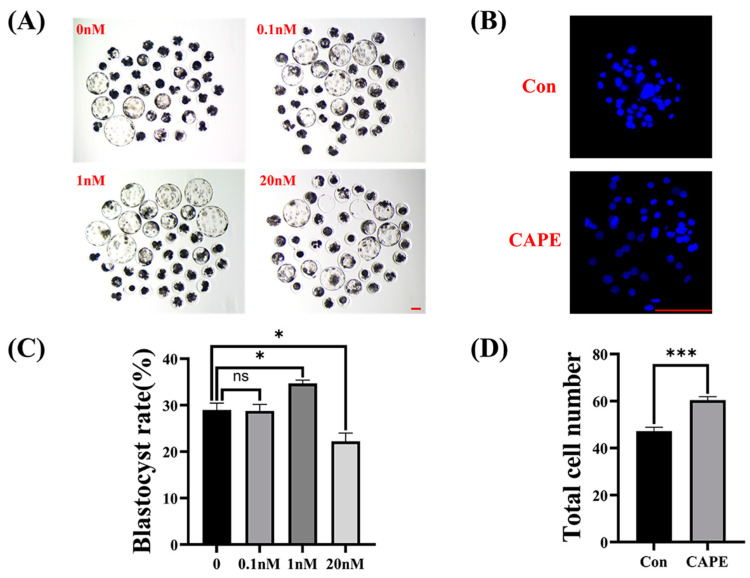
Effects of different concentrations of CAPE on the formation of blastocysts in vitro. (**A**) Effects of different CAPE concentrations (0, 0.1, 1, and 20 nM) on porcine blastocyst formation rate, especially 1 nM. (**B**) Stained images of total cell number in day 7 blastocysts with or without 1 nM CAPE treatment. (**C**) Effects of different CAPE concentrations (0, 0.1, 1, and 20 nM) on porcine blastocyst formation rate, especially 1 nM. Number of oocytes (n) used here was 131, 129, 127, and 127 for 0, 0.1, 1, and 20 nM of CAPE, respectively. (**D**) Total cell number in the control (n = 35) and 1 nM CAPE-treated (n = 30) groups. Scale bars = 100 μm. * *p* < 0.05, *** *p* < 0.001.

We added four concentrations of CAPE (i.e., 0, 0.1, 1, and 20 nM) to the culturing medium and found that blastocysts grown on 1 nM of CAPE formed at a significantly higher rate than those grown without CAPE. The blastocyst formation rates of the 0, 0.1, 1 and 20 nM groups were 28.97 ± 1.48%, 28.77 ± 1.43%, 34.67 ± 0.74%, and 22.21 ± 1.80% (Figure 1A,C). Although adding 0.1 nM or 20 nM of CAPE did not cause a significant increase in the blastocyst formation rate, the quality of blastocysts improved to some extent (e.g., 20 nM CAPE-promoted blastocyte hatching; Figure 1A). To further support the suitability of 1 nM as a treatment concentration for subsequent experiments, we also stained the control and 1 nM CAPE-treated blastocysts with Hoechst 33342 dye; the total cell number in the control group was lower than the 1 nM CAPE groups (47.23 ± 1.64 versus 60.43 ± 1.47, *** *p* < 0.001; Figure 1B,D). Based on these results, we concluded that 1 nM of CAPE is the most suitable concentration for the subsequent experiments. Therefore, in the subsequent experiments, the CAPE-treated group had a concentration of 1 nM of CAPE.

### 3.2. CAPE Improves Early Embryo Proliferation

**Figure 2:**
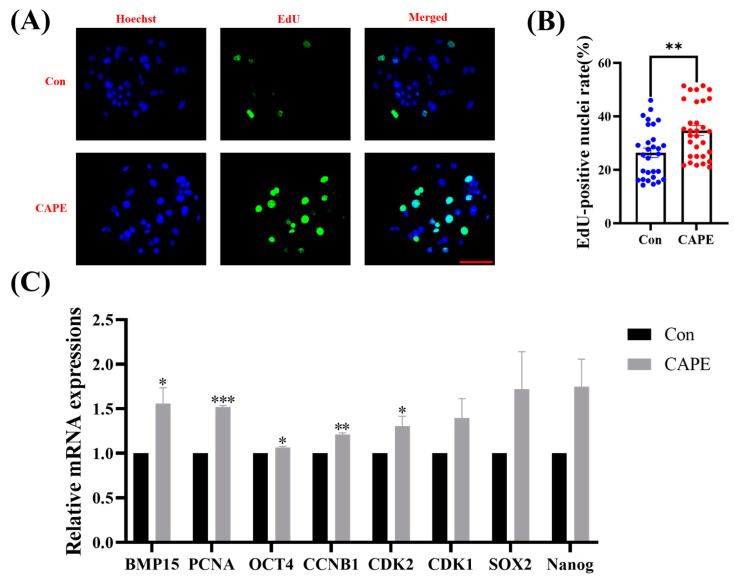
Effect of CAPE on the proliferative capacity of porcine embryos. (**A**) Representative 5-ethynyl-2′-deoxyuridine (EdU) staining images. The blue colour represents the nucleus and the green colour represents the EdU-positive nuclei. (**B**) Relative levels of proliferating cells in embryos treated with (n = 31) or without (n = 29) CAPE. (**C**) Relative mRNA expression levels of embryonic developmental genes, BMP15, PCNA, OCT4, CCNB1, CDK2, CDK1, SOX2, and NANOG, quantified with RT-qPCR. Scale bars = 100 μm. * *p* < 0.05, ** *p* < 0.01, *** *p* < 0.001.

We investigated the proliferative capacity of embryos and the expression of genes related to embryonic development and pluripotency to explore how 1 nM of CAPE promotes embryonic development. We discovered that the number of EdU-positive nuclei significantly increased in CAPE-treated embryos (34.70 ± 1.85%) compared to the control (26.35 ± 1.77%, ** *p* < 0.01; Figure 2A,B). Subsequently, we analyzed the mRNA expression levels of genes associated with embryonic development under the CAPE treatment. Remarkably, CAPE increased the expression of the following developmentally relevant genes: bone morphogenetic protein 15 (BMP15) (* *p* < 0.05), proliferating cell nuclear antigen (PCNA) (*** *p* < 0.001), octamer-binding transcription factor 4 (OCT4) (* *p* < 0.05), cyclin B1 (CCNB1) (** *p* < 0.01), and cyclin-dependent kinase 2 (CDK2) (* *p* < 0.05). Cyclin-dependent kinase 1 (CDK1), SRY-box transcription factor 2 (SOX2), and nanog homeobox (NANOG) showed no significant differences in expression (Figure 2C).

### 3.3. CAPE Enhances the Capacity of the Early Embryo Antioxidant System

**Figure 3:**
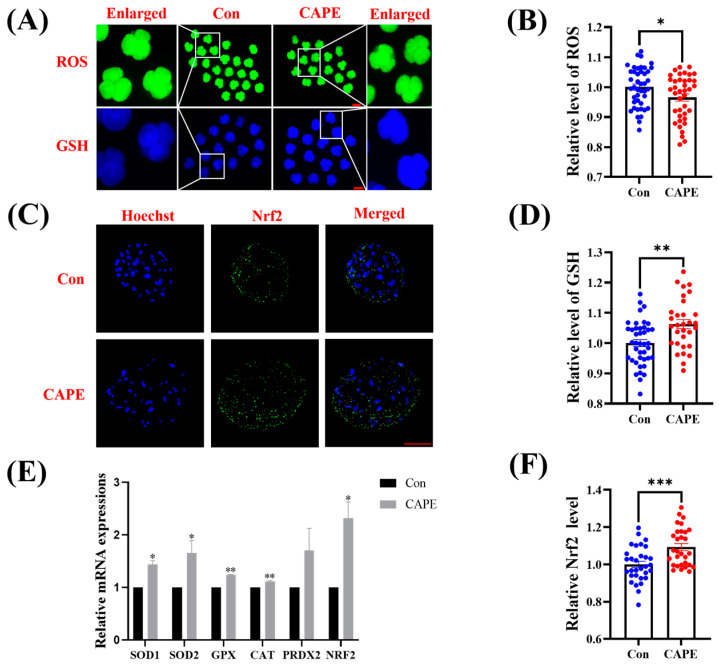
Effect of CAPE on the antioxidant performance of porcine embryos in vitro. (**A**) Representative 4-chloromethyl-6,8-difluoro-7-hydroxycoumarin and 2′,7′-dichlorodihydrofluorescein diacetate staining images. The green colour represents ROS and the blue colour represents GSH. (**B**) Relative changes in CMF2HC fluorescence intensity in the control (n = 43) and CAPE-treated (n = 39) groups. (**C**) Representative images of blastocysts stained for the Nrf2 protein. The blue colour represents the nucleus and the green colour represents the Nrf2 protein. (**D**) Relative changes in DCFH fluorescence intensity in the control (n = 33) and CAPE-treated (n = 32) groups. (**E**) Relative mRNA expression levels of antioxidant-related genes, SOD1, SOD2, GPX, CAT, PRDX2, and NRF2, quantified by RT-qPCR. (**F**) Fluorescence intensity of the Nrf2 protein in blastocysts treated with (n = 30) or without (n = 31) CAPE. Scale bars = 100 μm. * *p* < 0.05, ** *p* < 0.01, *** *p* < 0.001.

By staining porcine embryos with DCFH and CMF2HC, we found that those in the CAPE-treated group had significantly reduced intracellular ROS content (* *p* < 0.05; Figure 3A,B) and increased GSH levels (** *p* < 0.01; Figure 3A,D) versus those in the control group. We also performed immunofluorescence staining, revealing that the fluorescence intensity of the nuclear factor erythroid 2-related factor 2 (Nrf2) protein was significantly amplified in the embryos from the CAPE group, compared to the control group (*** *p* < 0.001; Figure 3C,F). Subsequently, we quantified mRNA expression levels of genes encoding antioxidant enzymes under CAPE treatment. We uncovered that CAPE enhanced the mRNA expression of superoxide dismutase 1 (SOD1) (* *p* < 0.05), SOD2 (* *p* < 0.05), glutathione peroxidase (GPX) (** *p* < 0.01), catalase (CAT) (** *p* < 0.01), and Nrf2 (* *p* < 0.05), but had no significant effect on peroxiredoxin 2 (PRDX2) (Figure 3E).

### 3.4. CAPE Effectively Improves Mitochondrial Function in Early Embryos

**Figure 4:**
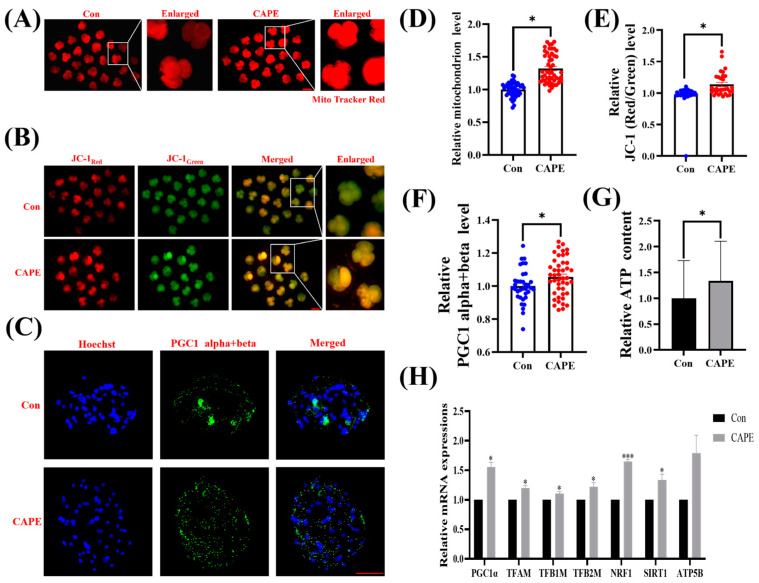
Effects of CAPE on mitochondrial function of porcine embryos. (**A**) Representative MitoTracker Red CMXRos staining images. The red colour represents the MitoTracker. (**B**) Representative JC-1 staining images. The red colour represents the JC1 red fluorescence, the green colour represents the JC1 green fluorescence and the orange colour represents the merged image of the two fluorescence channels. (**C**) Representative images of blastocysts with PGC1 alpha and beta proteins detected with immunofluorescence staining. The blue colour represents the nucleus and the green colour represents the PGC1 alpha and beta protein. (**D**) Relative fluorescence levels of MitoTracker Red in 4-cell-stage embryos treated with (n = 50) or without (n = 50) CAPE. (**E**) Relative fluorescence levels of JC-1 red/green in 4-cell-stage embryos treated with (n = 31) or without (n = 34) CAPE. (**F**) Relative fluorescence levels of PGC1 alpha and beta in blastocysts treated with (n = 45) or without (n = 35) CAPE. (**G**) Relative ATP content in blastocysts treated with (n = 60) or without (n = 60) CAPE. (**H**) Relative mRNA expression levels of mitochondrial function-related genes, PGC1α, TFAM, TFB1M, TFB2M, NRF1, SIRT1, and ATP5B, analyzed by RT-qPCR. Scale bars = 100 μm. * *p* < 0.05, *** *p* < 0.001.

MitoTracker Red CMXRos staining showed that 4-cell-stage embryos from the CAPE-treated group significantly increased mitochondrial abundance versus those in the control group (* *p* < 0.05; Figure 4A,D). Further, the evaluation of MMP in porcine embryos using JC-1 staining revealed a significant enhancement of MMP in the CAPE-treated group compared to the control group (* *p* < 0.05; Figure 4B,E). We also assessed the fluorescence intensity of embryos immunofluorescently stained with antibodies against PPARgamma coactivator 1alpha (PGC1α) and PGC1β proteins. Because these proteins showed significantly elevated levels in the embryos from the CAPE-treated group (* *p* < 0.05; Figure 4C,F), we concluded that CAPE promotes mitochondrial biogenesis and oxidative phosphorylation in the embryos. Indeed, when we quantified ATP levels in the embryos, we found significantly higher ATP levels in the CAPE-treated group than the control group (* *p* < 0.05; Figure 4G). Subsequently, we assessed how CAPE affects the mRNA expression of genes involved in the regulation of mitochondrial function. Our results revealed that CAPE increased the mRNA expression levels of PGC1α (* *p* < 0.05), transcription factor A, mitochondrial (TFAM) (* *p* < 0.05), transcription factor B1, mitochondrial (TFB1M) (* *p* < 0.05), TFB2M (* *p* < 0.05), nuclear respiratory factor 1 (NRF1) (*** *p* < 0.001), and sirtuin 1 (SIRT1) (* *p* < 0.05), but had no significant effect on ATP5B (ATP synthase f1 subunit beta) (Figure 4H).

### 3.5. CAPE Alleviates ER Stress in Early Embryos

**Figure 5:**
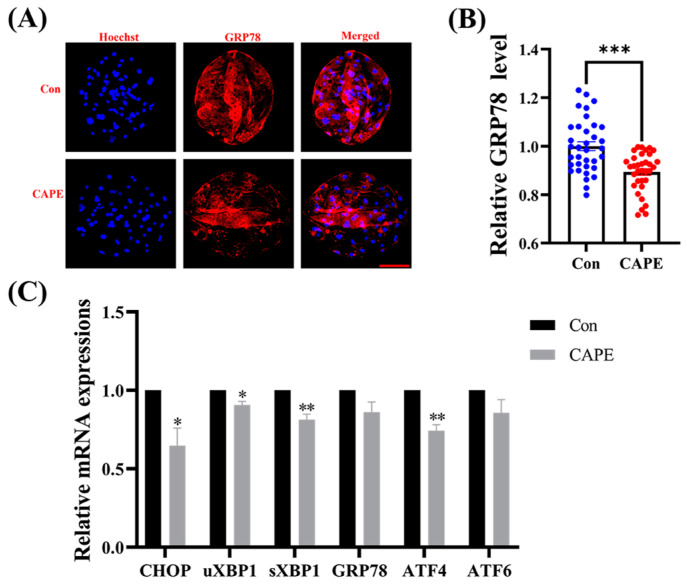
Effect of CAPE on endoplasmic reticulum stress of porcine embryos. (**A**) Representative images of blastocysts with the expression of the GRP78 protein determined with immunofluorescence staining. The blue colour represents the nucleus and the red colour represents the PGC1 alpha and beta protein. (**B**) Relative fluorescence levels of the GRP78 protein in blastocysts treated with (n = 34) or without (n = 35) CAPE. (**C**) Relative mRNA expression levels of ER stress-related genes, CHOP, uXBP1, sXBP1, GRP78, ATF4, and ATF6, quantified by RT-qPCR. Scale bars = 100 μm. * *p* < 0.05, ** *p* < 0.01, *** *p* < 0.001.

Our immunofluorescence staining results revealed that the expression of ER stress marker glucose-regulated protein 78kDa (GRP78) was lower in the CAPE-treated group (*** *p* < 0.001; Figure 5A,B). Subsequently, we assessed the mRNA expression levels of ER stress-associated genes in embryos exposed to CAPE. Our results uncovered that CAPE reduced the mRNA expression levels of C/EBP homologous protein (CHOP) (* *p* < 0.05), activating transcription factor 4 (ATF4) (** *p* < 0.01), the inactive form of X-box-binding protein 1 (uXBP1) (* *p* < 0.05), and its active form sXBP1 (** *p* < 0.01), but it did not affect GRP78 and ATF6 (Figure 5C).

### 3.6. CAPE Reduces Autophagy and Apoptosis in Embryos

**Figure 6:**
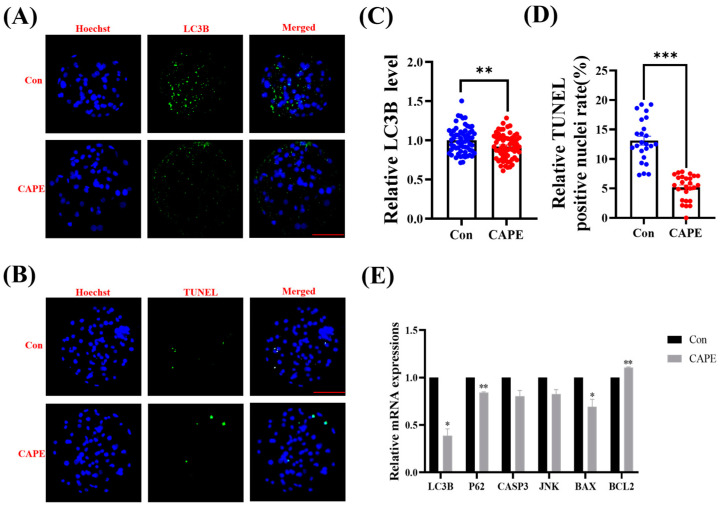
Effect of CAPE on autophagy and apoptosis of in vitro-produced early porcine embryos. (**A**) Representative immunofluorescence staining images of the LC3B protein in blastocysts. The blue colour represents the nucleus and the green colour represents the LC3B protein. (**B**) Representative images of blastocysts stained with TUNEL. The blue colour represents the nucleus and the green colour represents the apoptotic nuclei. (**C**) Relative autophagy levels in embryos treated with (n = 64) or without (n = 61) CAPE. (**D**) Relative apoptosis levels in embryos treated with (n = 26) or without (n = 24) CAPE on day 7. (**E**) Relative mRNA expression levels of autophagy and apoptosis marker genes, LC3B, P62, CASP3, JNK, BAX, and BCL2, quantified using RT-qPCR. Scale bars = 100 μm. * *p* < 0.05, ** *p* < 0.01, *** *p* < 0.001.

We immunostained blastocysts for the MAP1A/MAP1B Light Chain 3 B (LC3B) protein to assess whether CAPE affects autophagy levels in the embryos. Reduced LC3B staining indicated a significant reduction in autophagy levels in the CAPE-treated group compared to the control group (0.92 ± 0.20-fold, ** *p* < 0.01; Figure 6A,C). We also performed TUNEL staining to detect DNA breaks in blastocysts exposed to CAPE treatment. Significantly fewer DNA breaks in the CAPE-treated group compared to the control group (13.11 ± 0.75% versus 5.17 ± 0.42%, *** *p* < 0.001; Figure 6B,D) suggested reduced apoptosis under CAPE treatment. Subsequently, we assessed the mRNA expression levels of autophagy- and apoptosis-related genes to verify the effects of CAPE on autophagy and apoptosis. It was found that 1 nM of CAPE treatment reduced the expression of autophagy-related genes LC3B (* *p* < 0.05) and autophagy receptor P62 (P62) (** *p* < 0.01), which agrees with our staining results. It also decreased the expression of BCL2-associated x protein (BAX) (* *p* < 0.05), and increased the expression of anti-apoptotic gene BCL2 (BCL2 apoptosis regulator) (** *p* < 0.01). The treatment did not affect the expression of apoptosis-related genes caspase 3 (CASP3) and JUN N-terminal kinase (JNK) (Figure 6E).

## 4. Discussion

In recent years, the Chinese medicine industry has been revitalized, and there are many existing works on the extraction and separation of the components of Chinese medicine, as well as in-depth studies on the active ingredients. As a natural medicine, propolis has medicinal value, as well as being widely used as a biocosmetic and functional material [18]. Its active ingredient, CAPE, has been found to act against cancers, including prostate [19], colon [20,21], and gastric [22] cancers, through a variety of pathways. However, the potential of CAPE in promoting embryonic development remains unexplored. Based on the knowledge of the wide range of biological activities of CAPE, we are very interested in the potential of CAPE in reproductive medicine, especially in embryonic development.

In this study, we revealed that adding CAPE to the culture medium significantly increased the blastocyst formation rate and increased the total cell number. Furthermore, CAPE promoted the proliferative capacity of blastocysts and induced the mRNA levels of genes related to embryonic development. Among these genes were BMP15, an oocyte-specific factor expressed at higher mRNA levels in good-quality blastocysts, and OCT4, a core transcription factor maintaining pluripotency in the early mammalian embryo. Other notable genes were PCNA, a key regulator in many crucial cellular processes, and CCNB1 and CDK1/2, genes regulating the cell cycle. The CDK1/2 kinase may regulate early porcine embryo development, and CDK2 repression delays the division and developmental arrest of the embryo [23,24,25,26,27]. In addition, CAPE inhibits cyclin complexes in cancer cells and results in cell cycle arrest [28]. None of these results would be contrary to the results of the previous work on CAPE, and they are consistent with our assumptions about the likely effects of CAPE. In conclusion, our results suggest that CAPE may enhance embryo proliferation by increasing the mRNA expression levels of embryo development-related and cell cycle-associated genes.

External factors, such as cigarettes, ozone, and radiation, and internal factors, such as mitochondria-induced ROS accumulation, trigger cellular oxidative stress. The NF-kB protein is the first eukaryotic transcription factor to respond directly to oxidative stress, and CAPE protects from oxidative stress via its environment-dependent antioxidant activity [29,30,31,32,33,34]. We found that CAPE adjusts the balance of the antioxidant system by decreasing ROS accumulation and increasing GSH levels in the embryo. The Nrf2 protein induces the expression of various genes encoding cytoprotective proteins that translocate to the nucleus and bind to antioxidant response elements [35,36]. We experimentally demonstrated that CAPE influences the Nrf2-mediated regulatory network that affects mitochondrial function and unfolded protein responses in the embryo by enhancing Nrf2 gene and protein expression. Furthermore, the PRDX2 enzyme fine-tunes embryonic peroxide levels during in vitro production [37]. Experiments revealed that CAPE increased the expression of antioxidant-related genes such as PRDX2, SOD1/2, GPX, and CAT, as inferred from their mRNA levels. Thus, CAPE seems to modulate the performance of the embryonic antioxidant system of embryos produced in vitro. Then, when the embryos produced in vitro are subjected to various adverse effects caused by the external environment, CAPE is able to exert its antioxidant properties and increase the antioxidant capacity in porcine embryos in a molecular mechanism to protect the embryos from the occurrence of oxidative stress that may be induced by the adverse environment.

Mitochondria control the ATP production and redox state, acting as intracellular signal transduction elements. These organelles are thought to have similar abilities during mammalian oogenesis and early embryogenesis, where ATP production is the main energy source for cellular metabolism [38,39,40]. The PGC1α/β protein is a master regulator of mitochondrial biogenesis and function, inducing mitochondrial biogenesis through NRF1 activation [41]. Other proteins promoting mitochondrial biogenesis are the SIRT1 protein, a deacetylase that regulates PGC1α activity and is associated with mitochondrial biogenesis, and the TFAM, TFB1M, and TFB2M transcription factors, which control the expression of mitochondrial proteins. The ATP5B enzyme is a subunit of the mitochondrial ATP synthase that catalyzes ATP production during oxidative phosphorylation [42,43,44]. Our study demonstrated that CAPE increases the number of functional mitochondria, raises the levels of the mitochondrial membrane potential, and enhances PGC1 expression, which, in turn, regulates mitochondrial biogenesis and increases ATP production in mitochondria. Indeed, the elevated expression of many genes controlling the mitochondrial function (i.e., PGC1α, TFAM, TFB1M/2M, NRF1, SIRT1, and ATP5B) under CAPE treatment indicates that CAPE effectively enhances mitochondrial function. CAPE may then provide a stable source of energy to supply the embryos produced in vitro for subsequent life activities.

Mitochondria and the ER are essential, highly dynamic organelles in mammalian cells, and their interaction is a key hub for cellular stress. The processing, modification, and folding of proteins in the ER are cell-determining events [45,46,47]. Cells initiate the unfolded protein response (UPR) to mitigate ER stress triggered by unfavorable external stimuli, as high levels of ER stress provoke cellular damage. The UPR mechanism consists of three crucial ER transmembrane proteins, which are as follows: protein kinase RNA-like endoplasmic reticulum kinase, inositol-requiring protein 1, and ATF6 [48]. Other proteins involved in the ER stress response are CHOP, an ER stress protein, and GRP78, which forces unfolded proteins to refold or be degraded by cellular degradation mechanisms [49,50]. The existence of spliced (sXBP1, active) and unspliced (uXBP1, inactive) forms of XBP1 protein has an effective impact on the UPR, and exists in a spliced (sXBP1, active) or an unspliced form (uXBP1, inactive), as well as ER stress markers [51,52]. We showed that CAPE reduced GRP78 protein levels and decreased the mRNA levels of GRP78, CHOP, uXBP1, sXBP1, and ATF4/6, suggesting that CAPE alleviates ER stress in the embryo.

Animal cell death is typically classified as type I (apoptotic), type II (autophagic), or necrotic [53]. Mitochondria regulate calcium ion signaling, and calcium ion accumulation in mitochondria leads to apoptosis [54]. Autophagy, the process by which eukaryotic cells selectively dispose of misfolded, damaged, or excess cytoplasmic products, is induced under various stress conditions, such as ER stress [55,56,57]. Autophagy can also be understood as an environmental response to the cell’s call to act as a cleaner within the cell, and it is particularly important so that we can examine its occurrence in the cell as a good way to assess the quality of embryonic development. The LC3B protein is an RNA-binding protein that interacts with target mRNAs during autophagy, triggering their rapid degradation. It also attaches to autophagosomal membranes upon lipidation in the cytosol, recruiting P62, another autophagy marker with multiple functions, such as the NF-kB signaling pathway activation [58,59]. The CASP3 protein is a cysteine-aspartic acid protease that plays a central role in the execution phase of apoptosis [60]. In addition, the JNK kinase belongs to the MAPK family of kinases and is involved in apoptosis and the regulation of embryonic development [61]. Our results showed that CAPE suppressed autophagy in early embryos, which is made evident by the decreased LC3B protein and LC3B mRNA levels, and the decreased P62 mRNA levels. It also reduced the apoptosis rate of early embryos, and the mRNA levels of pro-apoptotic genes BAX, CASP3, and JNK. In contrast, CAPE increased mRNA levels of the anti-apoptotic marker BCL2, reinforcing the notion that CAPE represses autophagy and apoptosis in the in vitro-produced embryos.

## 5. Conclusions

**Figure 7:**
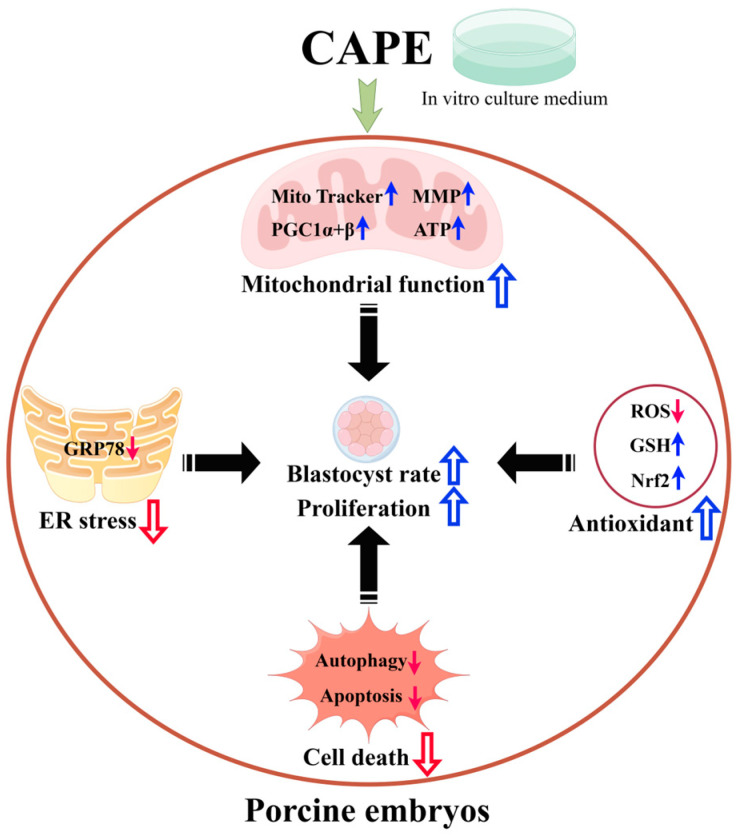
A schematic illustration summarizes how caffeic acid phenethyl ester (CAPE) improves the quality of in vitro-produced porcine embryos by affecting the mitochondrial function, ER stress, antioxidant performance, and cell death. Treating early embryos with 1 nM of CAPE enhances mitochondrial function by increasing the number of functional mitochondria, elevating the mitochondrial membrane potential, and enhancing PGC1 alpha and beta protein expression, which, in turn, promotes mitochondrial biogenesis and increases mitochondrial ATP production. In addition, the CAPE treatment enhances antioxidant properties by lowering ROS production and increasing GSH content and Nrf2 expression. The CAPE treatment also alleviates ER stress by reducing GRP78 expression and hindering autophagy and apoptosis. Solid blue arrows in the figure indicate an increase in relevant content or expression, and blue hollow arrows indicate a facilitation or improvement in relevant function. Solid red arrows in the figure indicate a decrease in the relevant content or expression, and red hollow arrows indicate that the relevant response was inhibited. (By Figdraw, https://www.figdraw.com, 19 January 2024).

In conclusion, 1 nM of caffeic acid phenethyl ester (CAPE) increased the rate of blastocyst formation and enhanced the proliferation of in vitro-produced porcine embryos by affecting cellular organelles, including the enhancement of the mitochondrial function and the alleviation of endoplasmic reticulum-triggered endoplasmic reticulum stress, cell death—including the reduction of apoptosis and autophagy—and the cellular antioxidant capacity in the embryo. Our findings can further enrich the application of propolis and CAPE in food and drug development, and help to improve the conditions for in vitro culture and the production of embryos, which will be useful for further applications, such as assisted reproduction technology (Figure 7).

## Figures and Tables

**Table 1:** Primer sequences used for RT-qPCR.

No.	Genes *	Primer Sequences (5′-3′)	Base
1	RN18S	F: TCCAATGGATCCTCGCGGAA R: GGCTACCACATCCAAGGAAG	20 20
2	BAX	F: TGCCTCAGGATGCATCTACC R: AAGTAGAAAAGCGCGACCAC	20 20
3	BCL2	F: AGGGCATTCAGTGACCTGAC R: CGATCCGACTCACCAATACC	20 20
4	BMP15	F: CCTCCATCCTTTCCAAGTCA R: GTGTAGTACCCGAGGGCAGA	20 20
5	CDK1	F: GGGCACTCCCAATAATGAAGT R: GTTCTTGATACAACGTGTGGGAA	21 23
6	CDK2	F: TGTGCGAGTGGATGCGGAAG R: CCGAATGGTGATGTAGCGAC	20 20
7	NANOG	F: GGTTTATGGGCCTGAAGAAA R: GATCCATGGAGGAAGGAAGA	20 20
8	SOX2	F: ATGCACAACTCGGAGATCAG R: TATAATCCGGGTGCTCCTTC	20 20
9	TFAM	F: CCTCTGTGCGGTTTGTGGAAGTC R: TACACCTGCCAGTCTGCCCTATAAG	23 25
10	NRF1	F: GCCGATGCTTCAGAATTGCCAAC R: TCCACCTCTCCATCAGCCACAG	23 22
11	NRF2	F: AGCGGATTGCTCGTAGACAG R: TTCAGTCGCTTCACGTCGG	20 19
12	TFB1M	F: CCCAAGATAGAGCAGCCGTTCAAG R: CCAAGCCCTCGATAGCAGTATTTCC	24 25
13	TFB2M	F: GCGGCAAGGAGGAAGGATGTTC R: GCACCAAGTTCTCAGCCACTCTC	22 23
14	PGC1α	F: AGGGAGAGGCAGAGGCAGAAG R: TGTCCGTGTTGTGTCAGGTCTG	21 22
15	ATF4	F: AGTCCTTTTCTGCGAGTGGG R: CTGCTGCCTCTAATACGCCA	20 20
16	uXBP1	F: CATGGATTCTGACGGTGTTG R: GTCTGGGGAAGGACATCTGA	20 20
17	sXBP1	F: GGAGTTAAGACAGCGCTTGG R: GAGATGTTCTGGAGGGGTGA	20 20
18	ATF6	F: TACTTCCAGCAGCACCCAAG R: GCACCACCGTCTGACCTTTA	20 20
19	CHOP	F: CCCCTGGAAATGAGGAGGAG R: CTCTGGGAGGTGTGTGTGAC	20 20
20	GRP78	F: GCTCTACTCGCATCCCCAAAG R: TACACCAGCCTGAACAGCAG	21 20
21	JNK	F: CTCGCTACTACAGAGCACCTG R: TTCTCCCATAATGCACCCCAC	21 21
22	CCNB1	F: CCAACTGGTTGGTGTCACTG R: GCTCTCCGAAGAAAATGCAG	20 20
23	SOD1	F: CTCTCGGGAGACCATTCCATCATTG R: TCCACCTCTGCCCAAGTCATCTG	25 23
24	SOD2	F: TGTATCCGTCGGCGTCCAAGG R: TCCTGGTTAGAACAAGCGGCAATC	21 24
25	SIRT1	F: CGGCAGGAGAAGGAAACAATGGG R: TCGTCGTCGTCGTCGTCGTAG	23 21
26	CAT	F: AGCCAGTGACCAGATGAAGCATTG R: ATGTCGTGTGTGACCTCAAAGTAGC	24 25
27	GPX	F: CTGGTCGTGCTCGGCTTCC R: GCCTGGTCGGACGTACTTGAG	19 21
28	CASP3	F: CGTGCTTCTAAGCCATGGTG R: GTCCCACTGTCCGTCTCAAT	20 20
29	LC3B	F: TTCAAACAGCGCCGAACCTT R: TTTGGTAGGATGCTGCTCTCG	20 21
30	P62	F: GACAACTGTTCAGGAGGAGACGATG R: AGAGACTGGAGTTCACCTGTAGACG	25 25
31	OCT4	F: GTGAGAGGCAACCTGGAGAG R: TCGTTGCGAATAGTCACTGC	20 20
32	PCNA	F: CCTGTGCAAAAGATGGAGTG R: GGAGAGAGTGGAGTGGCTTTT	20 21
33	PRDX2	F: TGTCCTTCGCCAGATCACT R: TCCACGTTGGGCTTGATT	19 18
34	ATP5B	F: TTGTTGGCAGTGAGCATT R: AACCTGGAATGGCTGAGA	18 18

* Housekeeping gene: RN18S; developmentally relevant genes: BMP15, PCNA, OCT4, CCNB1, CDK2, CDK1, SOX2, NANOG; antioxidant-related genes: SOD1, SOD2, GPX, CAT, NRF2, PRDX2; mitochondrial function-related genes: PGC1α, TFAM, TFB1M, TFB2M, NRF1, SIRT1, ATP5B; ER stress-related genes: CHOP, uXBP1, sXBP1, ATF4, GRP78, ATF6; autophagy-related genes: LC3B, P62; apoptosis-related genes: BAX, BCL2, CASP3, JNK.

## Data Availability

The data underlying this article will be shared upon reasonable request to the corresponding author.
